# Albino lethal 13, a chloroplast‐imported protein required for chloroplast development in rice

**DOI:** 10.1002/pld3.610

**Published:** 2024-06-20

**Authors:** Xiaoqiong Guo, Chunli Wang, Qian Zhu, Wenhua Dongchen, Xiaoling Zhang, Wei Li, Hui Zhang, Cui Zhang, Zar Ni Naing Nant Nyein, Mengting Li, Lijuan Chen, Dongsun Lee

**Affiliations:** ^1^ Rice Research Institute Yunnan Agricultural University Kunming China; ^2^ College of Biological Resource and Food Engineering Qujing Normal University Qujing China; ^3^ State Key Laboratory for Conservation and Utilization of Bio‐Resources in Yunnan Yunnan Agricultural University Kunming China; ^4^ The Key Laboratory for Crop Production and Smart Agriculture of Yunnan Province Yunnan Agricultural University Kunming China; ^5^ College of Agronomy and Biotechnology Yunnan Agricultural University Kunming China; ^6^ Kunming University Kunming China

**Keywords:** albino gene, chloroplast development, *OsAL13*, rice ( *Oryza sativa* L.)

## Abstract

Chloroplasts play a vital role in plant growth and development, which are the main sites of photosynthesis and the production of hormones and metabolites. Despite their significance, the regulatory mechanisms governing chloroplast development remain unclear. In our investigation, we identified a rice mutant with defective chloroplasts in rice ( *Oryza sativa* L.), named albino lethal 13 (*osal13*), which displayed a distinct albino phenotype in leaves, ultimately resulting in seedling lethality. Molecular cloning revealed that *OsAL13* encodes a novel rice protein with no homologous gene or known conserved domain. This gene was located in the chloroplast and exhibited constitutive expression in various tissues, particularly in green tissues and regions of active cell growth. Our study's findings reveal that RNAi‐mediated knockdown of *OsAL13* led to a pronounced albino phenotype, reduced chlorophyll and carotenoid contents, a vesicle chloroplast structure, and a decrease in the expression of chloroplast‐associated genes. Consequently, the pollen fertility and seed setting rate were lower compared with the wild type. In contrast, the overexpression of *OsAL13* resulted in an increased photosynthetic rate, a higher total grain number per panicle, and enhanced levels of indole‐3‐acetic acid (IAA) in the roots and gibberellin A3 (GA3) in the shoot. These outcomes provide new insights on the role of *OsAL13* in regulating chloroplast development in rice.

## 1 INTRODUCTION

Nowadays, photosynthesis has become a major target of international consortia to increase yield potential (Furbank et al., 2023). Chloroplasts are major organelles for photosynthesis, which not only convert light energy into chemical energy but also synthesize biologically essential compounds (Kirchhoff, 2019). Therefore, the normal development and formation of chloroplasts are essential for the growth and development of plants and algae.

In general, chloroplasts develop from proplastids, a small group of colorless, differentiated plastids that exist mainly in the zygote, apical meristem, or newly created cells in mitosis. Under light conditions, the inner membrane of the proplastids in young leaves folds in many places, extends into the matrix, gradually expands and increases, and finally breaks away from the inner membrane to form a flat cystoid structure—a thylakoid. Many thylakoids stack together to form chloroplast grana, from which the proplastids gradually develop into mature chloroplasts (Kirchhoff, 2019; Lin et al., 2011).

Mutants of leaf color can be derived from a wide range of sources, including spontaneous, artificially induced, insertion, and gene silencing mutations (Hayashi‐Tsugane et al., 2014; Zeng et al., 2017; Zhang et al., 2018). Yoo et al. (2009) divided rice leaf phenotypes into types, including green leaf, albino, stripe, retarded green, zebra stripe, yellow, and variegated color. Li et al. (2018) classified rice leaf color mutations into light green, yellow, albino, and reversible green albino. Li et al. (2022) classified typical leaf color mutant phenotypes as albino, green, light green, striped, white, yellow, green, and yellow types. According to the characteristics of the generation and development of albinism, the albino phenotypes can be further divided into lethal albino, temperature‐sensitive regreening albino, reproductive regreening albino, and so forth. When the leaf becomes colored, the levels of growth‐promoting phytohormones, such as auxin, cytokinin (CTK), and gibberellin, are reduced (Lee & Masclaux‐Daubresse, 2021). Abnormal chloroplast development in cells also leads to abnormal cell division. In Arabidopsis *Atcls8* mutants, inhibition of chloroplast DNA replication leads to abnormal growth: leaf albinism, curling, and reduced root length (Garton et al., 2007).

Albino mutants in rice (*Oryza sativa* L.) are important genetic resources for studying genes related to chlorophyll biosynthesis, chloroplast development, and photosynthesis, as well as karyoplasmic interactions (Sheng et al., 2014). According to the characteristics of the generation and development of albinism, the albino phenotypes can be further divided into lethal albino, temperature‐sensitive regreening albino, reproductive regreening albino, and so forth. Lethal albino refers to the albino phenotype that begins with germination and then fails to return to green, and the three‐ or four‐leaf seedling plant dies after nutrition depletion. For example, the *albino seedling lethality 2* (*ASL2*) gene cloned in rice *asl2* mutant encodes the chloroplast 50S ribosomal protein L21 (RPL21C), which is a component of the chloroplast ribosome large subunit, and the lack of ASL2 function leads to chloroplast developmental defects and seedling death (Lin et al., 2015). Rice *lethal albino seedling 1* mutant developed an albino lethal phenotype due to 4‐hydroxy‐3‐methylbut‐2‐enyl diphosphate reductase deficiency (Liu et al., 2020). The *Oscaf2* mutant was obtained by gene editing in cv. Nipponbare; the *OsCAF2* mutation led to a lethal albino phenotype at the seedling stage (Shen et al., 2020). The rice genes *OsPPR1*, *OsPPR6*, *OsDjA 7/8*, *OsalS3*, *Ososptac2*, and *ALS1* (Gothandam et al., 2005; Lin et al., 2015; Tang et al., 2017; Wang et al., 2016; Xu et al., 2024; Zhu et al., 2015) and *Arabidopsis thaliana* genes *Atasd* and *Atskl1–8* (Xu et al., 2018) showed early chloroplast development defects leading to albino leaves.

In this study, a rice albino mutant *osal13* was screened from a T‐DNA insertion mutant library, and its genetic characteristics were analyzed by genetic population construction. The data showed that the albino trait was controlled by a single recessive nuclear gene. The target gene and its promoter were cloned, and its function was predicted using bioinformatics. The spatiotemporal expression pattern and genetic function of the target gene were studied by constructing four different expression vectors. β‐Glucuronidase (GUS) staining and quantitative real‐time polymerase chain reaction (qRT‐PCR) were used to reveal the expression pattern of the target gene. The number and ultrastructure of chloroplasts in mutants and RNAi were analyzed using fluorescence microscopy and transmission electron microscopy (TEM). The hormone contents in wild‐type (WT), *osal13* mutant, and transgenic plants were determined by enzyme‐linked immunosorbent assays to explore the regulatory effect of *OsAL13* on hormones. Multiple chloroplast‐associated genes were investigated. Overall, this study improved our understanding of the OsAL13 protein and revealed additional information about the molecular mechanism of *OsAL13* action in rice chloroplast development.

## 2 MATERIALS AND METHODS

### 2.1 Plant materials and growth conditions

The *O. sativa* *japonica* cv. LiyuB was used as the WT. The T‐DNA insertion library with a LiyuB background was obtained from the Rice Research Institute of Yunnan Agricultural University. Because the albino mutant died at the four‐leaf seedling stage, individual plants were harvested to preserve the seeds of the heterozygote. All rice seeds were propagated under natural growing conditions in a tightly controlled greenhouse at the Rice Research Institute of Yunnan Agricultural University.

### 2.2 Analysis of the T‐DNA insertion locus in the *osal13* mutant

Genomic DNA of the *osal13* albino mutant (MT) after germination 1 weeks was extracted by the cetyltrimethylammonium bromide (CTAB) method. DNA walking was performed using the DNA Walking Speedup™ Premix Kit (http://www.seegene.com). The target region was obtained by the first round of PCR amplification with the highly specific renaturation control primers (DW‐ACP2) and the target‐specific primers (Bar_TSP1). The first round of PCR products was used as the template, and the second round of amplification was carried out by the DW‐ACPN and the target‐specific primer (Bar_TSP2) to narrow the target area. Finally, the second round of PCR products was used as the template, and the third round of amplification was carried out by the universal primer (Unip2) and the new target‐specific primer (Bar_TSP3) to obtain the different fragments. Primers for testing of the T‐DNA inserting locus were Unip2 for the left site and Bar_TSP3 for the right. Primer sequences are listed in Table 1.

**TABLE 1:** List of primer sequences used in this study.

Primer name	Primer sequence (5′–3′)	Tm	Primer function
Bar_TSP1	ATGCACGAGGCGCTCGGATA		Test *al13* flanking of T‐DNA
Bar_TSP2	TCAAGCACGGGAACTGGCAT		
DW3ACP1	GAGGAGTGGCAGTGGGAACGGG	60°C	
DW3ACP2	GAGGAGTGGCAGTGGGCTCGA	60°C	
DW3ACP3	GAGGAGTGGCAGTGGGCTACG	60°C	
DW3ACP4	GAGGAGTGGCAGTGGGAACGG	60°C	
T‐DNA right border	TGTTTACACCACAATATATCCTGCCA		
T‐DNA left border	TGGCAGGATATATTGTGGTGTAAACA		
UniP2	GAGTTTAGGTCCAGCGTCCGT		
Bar_TSP3	TGCCCGTCACCGAGATCTGA		
OsAL13p‐F	GAATTCGGATTCGTCCGTTGCCATGA	61°C	Promoter cloning
OsAL13p‐R	CTGCAGGAGGCCAAGGTGGAGTTGG		
OsAL13‐1Fa	CTGCAGGACGGCCAAGGCGTGCAG	64°C	*OsAL13* full‐length ORF amplification
OsAL13‐1Ra	ACGCGTAGGGCGGATACCAAGTTCAACACGA		
OsAL13Ri‐1F	CATCCCCCGAAATCAGGTTGG	57°C	RNAi1 short fragment amplification
OsAL13Ri‐1R	GGGCGGATACCAAGTTCAACAC		
OsAL13Ri‐2F	ATCCGGCGTCTTAGTTCTAGCC	58°C	RNAi2 short fragment amplification
OsAL13Ri‐2R	GTGTTGAGGGAGCCAGGAAATC		
OsAL13RT‐F	CTCATGGTTTGTCGCCTTCGTC	58°C	qRT‐PCR
OsAL13RT‐R	GGCGGATACCAAGTTCAACACG		
HmMAS‐F	AGACCTGCCTGAAACCGAACTG	60°C	Transgenic plant screening
HmMAS‐R	TGTTGGCGACCTCGTATTGGGA		
β‐Actin‐F	CCGAGCGGGAAATTGTGAGGGA	62°C	qRT‐PCR housekeeping gene
β‐Actin‐R	TTTCAGGAGGGGCGACCACCTT		
psaA‐F	TTAGAAATCCGCCAATCCA	53°C	Reverse transcription PCR Plastid‐encoded polymerases (PEPs) refer to Zhang et al. (2016)
psaA‐R	TGCTAGGCTCTACAACCATT		
psaB‐F	GAGCAATATCGGTCAGCCACA	56°C	
psaB‐R	ACCACTCAAGGAGCGGGAAC		
psbA‐F	ACCCTCATTAGCAGATTCGT	57°C	
psbA‐R	GATTGTATTCCAGGCAGAGC		
rps14‐F	TCACTCAAACTCAAAGGGTA	53°C	
rps14‐R	AAGCGGCAGAAATTAGAAC		
atpA‐F	TATCGGTCAAAGAGCATC	58°C	Reverse transcription PCR Nucleus‐encoded polymerases (NEPs) refer to Zhang et al. (2016)
atpA‐R	CGTATAAGGAGCGAGGTA		
petA‐F	TGCCATTTAGCGAATAAGCC	57°C	
petA‐R	CCACATTCAACCCTCCCTTT		
rpoB‐F	TGGTACATATCCCTTATCTCAA	53°C	
rpoB‐R	CTCCAGGACCCAAACAACTC		
rps2‐F	GAGATGATAGAAGCGGGAGTT	55°C	
rps2‐R	TAACATAATGACAACGAGCC		

Abbreviations: ORF, open reading frame; qRT‐PCR, quantitative real‐time polymerase chain reaction.

### 2.3 Gene cloning, characterization, and bioinformatic analysis

Sequence alignment analysis showed that the T‐DNA was inserted into the *Os11g0307600* gene. Two different transcripts, Os11t0307600‐01 and Os11t0307600‐02, encoding 128 and 522 amino acids, respectively, were found at this gene site by RAP‐BD database retrieval. Primer 5 software was used to design primers OsAL13‐1F/‐1Ra and OsAL13‐1F/‐2Rb on both sides of the predicted gene region, and PCR amplification was performed using LiyuB cDNA as the template.

The *OsAL13* gene was annotated according to the full‐length cDNA sequence on the National Center for Biotechnology Information (NCBI) database. Sequence similarity was determined using the BLAST program (http://www.ncbi.nlm.nih.gov/BLAST/) provided by the NCBI website. Protein conserved domain analysis was performed using CD databases (https://www.ncbi.nlm.nih.gov/Structure/cdd/wrpsb.cgi).

### 2.4 Vector construction and transformation

The corresponding primers (Table 1) were amplified with the genomic DNA of LiyuB as the template to obtain the 2.3‐kb DNA fragment of the promoter region of *OsAL13*, which was confirmed by DNA sequencing and then ligated into the binary vector HPE193‐2. In order to obtain the overexpressing (OVE) plants, the corresponding primers were used to amplify a 610‐bp DNA fragment including the full open reading frame (ORF) of *OsAL13* with LiyuB cDNA as the template, which was confirmed by DNA sequencing and then ligated into the binary vector HPE192‐1. To construct the *OsAL13* RNAi vector (*p35S×2::OsAL13‐RNAi*), a 117‐bp intron fragment was used as a linker between a 154‐bp gene‐specific fragment (a 418‐bp gene‐specific fragment was generated synchronously) in the antisense and sense orientations. These recombined fragments were inserted into the HPE203‐1 binary vector containing a double 35S promoter.

The final constructs were transferred into *Agrobacterium tumefaciens* strain EHA105 using the freeze–thaw method for rice genetic transformation. The rice transformations were conducted as described by Toki et al. (2006). Eight OVE plants (*p35S*::*OsAL13:EGFP*), 12 *GUS* stained plants (*pAL13::GUS*), 11 RNAi1 plants (*p35S×2::OsAL13:RNAi1*), and 11 RNAi2 plants (*p35S×2::OsAL13:RNAi2*) were obtained in the T_0_ generation.

### 2.5 Histological GUS assay

The *GUS* activity analysis was performed following a standard protocol (Jefferson et al., 1987). Tissues of rice at different periods were taken, cut into appropriate sizes, and immediately immersed in 90% acetone at −20°C for 15–20 min. Acetone was removed, and the sample was rinsed with the following rinse solution: .1 M of K_3_Fe(CN)_6_, .1 M of K_4_Fe(CN)_6_, and .5 M of NAPO_4_ at pH 7.2. The rinse solution was removed, and X‐GluC staining solution (10% Triton X‐100, 20 mM of X‐GluC, .1 M of K_3_Fe(CN)_6_, .1 M of K_4_Fe(CN)_6_, and .5 M of NAPO_4_ at pH 7.2) was added in a test tube to ensure that the sample was completely immersed in the dye solution. At room temperature, the sample was vacuumed for 10–20 min to sink below the liquid level. The samples were treated overnight at 37°C. The samples were rinsed with 70% ethanol to remove the chlorophyll in the leaves. The cleaned samples were observed under an anatomical lens, and stained samples were photographed using a Nikon digital camera and further sliced using frozen sections (Leica CM1950, Leica Biosystems Nussloch GmbH, Nussloch, Germany) and analyzed under a dissecting microscope.

### 2.6 Total RNA extraction and qRT‐PCR analysis of gene expression

To explore the *OsAL13* expression pattern and level, total RNA was extracted from plant tissues at different developmental stages using a RNeasy Plant Mini Kit (Qiagen) according to the manufacturer's instructions. The first strand of cDNA was synthesized using the Revert Aid First Strand cDNA Synthesis Kit (Thermo Fisher) according to the manufacturer's protocol. All primers used in qRT‐PCR are listed in Table 1. The relative expression level of the target gene was normalized to that of rice *β‐actin*. qRT‐PCR was performed using a Super Real Premix Plus (SYBR Green) (Cat. No. FP205‐01; Tiangen Co, Ltd, Beijing, China) according to the instructions. The relative quantification values were calculated using the 2^−∆∆Ct^ method (Arocho et al., 2006), and three independent biological replicates were analyzed.

### 2.7 Confocal microscopy

To identify the subcellular localization of *OsAL13*, the ORF of the target gene and the 610‐bp fragment in the 5′UTR region of the target gene were successfully transferred into the enhanced green fluorescent protein (EGFP) vector HPE192‐1 (preserved in our laboratory), and the corresponding transgenic plants were obtained by the *Agrobacterium*‐mediated genetic transformation method into the embryogenic callus of rice LiyuB. The protoplasts of the transgenic *p35S::OsAL13: EGFP* and *p35S: EGFP* were extracted as described by Jiang et al. (2018).

### 2.8 Endogenous hormone assay

In order to clarify whether the *OsAL13* gene is involved in endogenous hormone regulation for plants, we measured the auxin (indole‐3‐acetic acid [IAA]), gibberellin A3 (GA3), and CTK contents of the WT, *OsAL13* MT, OVE, RNAi1, and RNAi2 plants at 10 days after germination (DAG). Endogenous contents of IAA, GA3, and CTK were determined as described by Zhu et al. (2017).

### 2.9 Chlorophyll detection

During the seedling stage, fresh leaves were collected and sliced into small pieces. Of these, about .05 g was placed in a 15‐mL tube, and 10 mL of 80% acetone was added. These were mixed in darkness for about 48 h (chlorophyll degrades under light). The extract was transferred to a 25‐mL volumetric flask, diluted with 80% acetone to volume, and thoroughly mixed. Then, chlorophyll content was determined using absorbance (A = 663, 646, and 470 nm). The chlorophyll concentrations were calculated as follows (use 80% acetone as a blank control):

$$\text{Chla}\;\left(\mathrm{mg}/g\right)=\left[12.21\times A663-2.81\times A646\right]\times V/\left(1000\times W\right),$$

$$\text{Chlb}\;\left(\mathrm{mg}/g\right)=\left[20.13\times A646-5.03\times A663\right]\times V/\left(1000\times W\right),$$

$$\text{Chla}+b\;\left(\mathrm{mg}/g\right)=\left[7.18\times A663+17.32\times A646\right]\times V/\left(1000\times W\right),$$

$$\beta -\mathrm{Car}\;\left(\mathrm{mg}/g\right)=\left[\left(1000\times A470-3.27\times \text{Chla}-104\times \text{Chlb}\right)/229\right]\times V/\left(1000\times W\right),$$

where V is volume of extract (milliliters) and W is weight of fresh leaves (grams).

### 2.10 Phenotype characterization

To explore the effect of target genes on the biological characteristics of rice, we measured the plant height, effective tiller, panicle length, seed setting rate, and primary and secondary branches of the WT and RNAi plants. Among them, plant height and effective tillers of each line were determined on eight plants, with plant height measured from the soil surface to the highest panicle top. Data were analyzed using PRISM 7.0 for one‐way analysis of variance (ANOVA) and Dunnett's multiple comparison test (*p < .05, **p < .01, and ***p < .001).

### 2.11 Statistical analysis

The original data were compiled using MS Excel 2019. All primers were designed using Primer Premier 5 software. All data were analyzed using GraphPad Prism 8.0 for one‐way ANOVA and Dunnett's multiple comparison test (*p < .05, **p < .01, and ***p < .001).

## 3 RESULTS

### 3.1 Phenotypic and physiological characteristics of the *osal13* mutant

**FIGURE 1:**
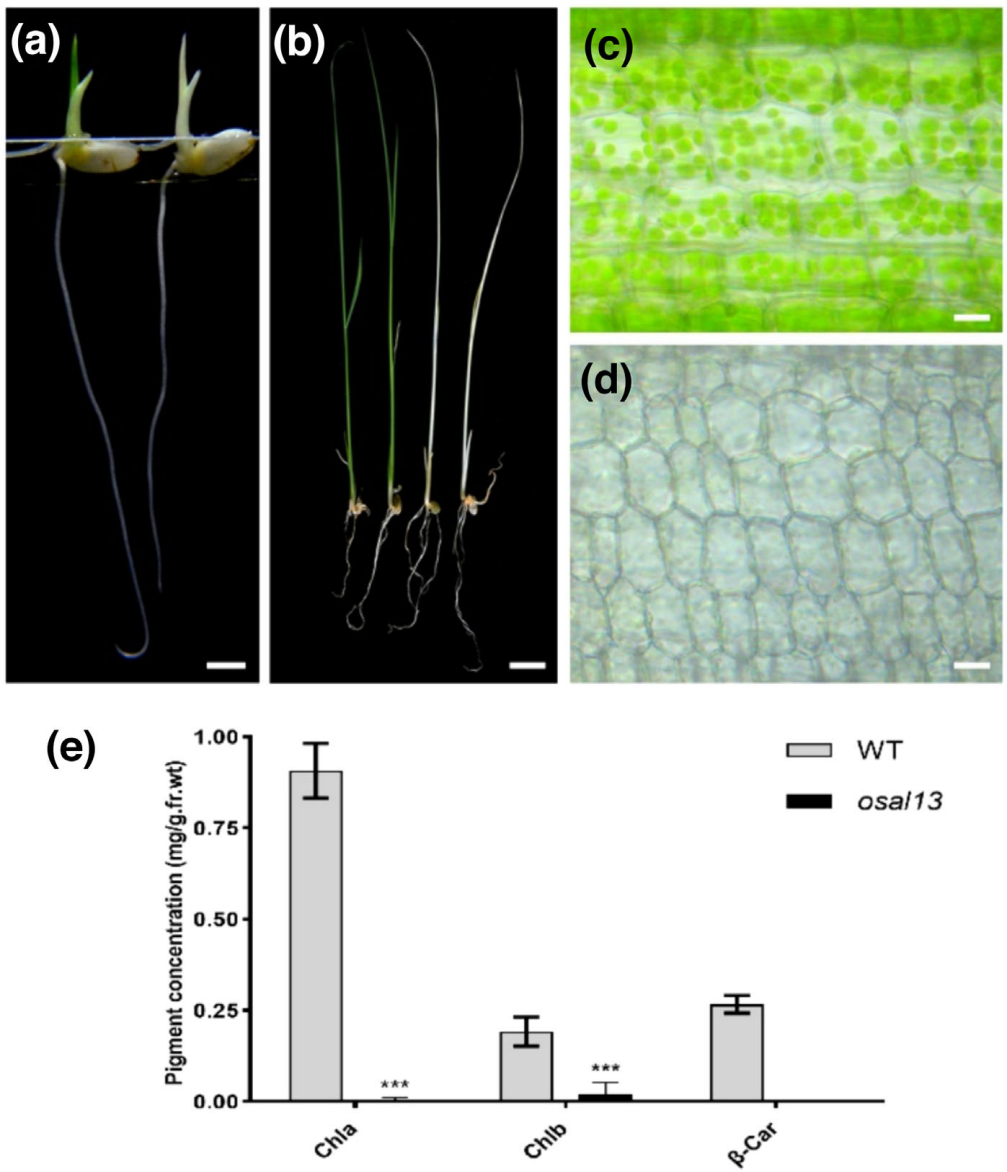
Mutations in the *OsAL13* gene affect the growth of rice and chloroplast development. (a, b) Phenotype analysis of the wild type (WT) and *osal13* mutant at 4 days after germination (DAG) and 20 DAG. Bars = 1 cm. (c, d) The sheath of WT and *osal13* mutant visualized using bright‐field microscopy. The scale is 25 μm. (e) Determination of pigment content in leaves of WT and *osal13* mutant. Leaf samples were collected from 20‐day‐old WT and *osal13* mutant grown in a tank containing nutrient solutions. Chla, chlorophyll a; Chlb, chlorophyll b; β‐Car, β‐carotenoid. Error bars are the standard deviation (SD) for three biological duplicates. Significant differences are indicated by asterisks (***p < .001).

The *osal13* mutant was a T‐DNA insertion mutation derived from plasmid vector pCAMBIA3300 transferred into japonica cv. LiyuB. Under light conditions, at 2 DAG, the WT and *osal13* mutant showed the same phenotype. However, they could be partially distinguished at 3 DAG. At 4 DAG, the first true leaf that grew in the bud sheath could be completely distinguished (Figure 1a). Although it exhibited a more severe albino phenotype during early leaf development, the severe albino phenotype of the *osal13* mutant eventually resulted in a lethal phenotype when they reached the four‐leaf stage (Figure 1b). Thus, we concluded that *OsAL13* is required for rice development.

With a view to verifying whether the *osal13* mutant was involved in chloroplast development, fresh sheath tissues were observed under bright‐field microscopy (Figure 1c,d), which revealed that cells of *osal13* mutant cotyledons had an apparent lower density of chloroplasts than WT cotyledons. This suggested a decreased chlorophyll content of the *osal13* mutant, in line with the pale green pigmentation of the *osal13* mutant compared with WT chloroplasts. Therefore, chlorophyll and carotenoid contents were measured in WT and *osal13*. As expected, levels of both chlorophylls and carotenoids were significantly lower in the *osal13* mutant (Figure 1e), demonstrating that the albino phenotype was caused by abnormal chlorophyll metabolism.

### 3.2 *OsAL13* cloning, characterization, and bioinformatic analysis

**FIGURE 2:**
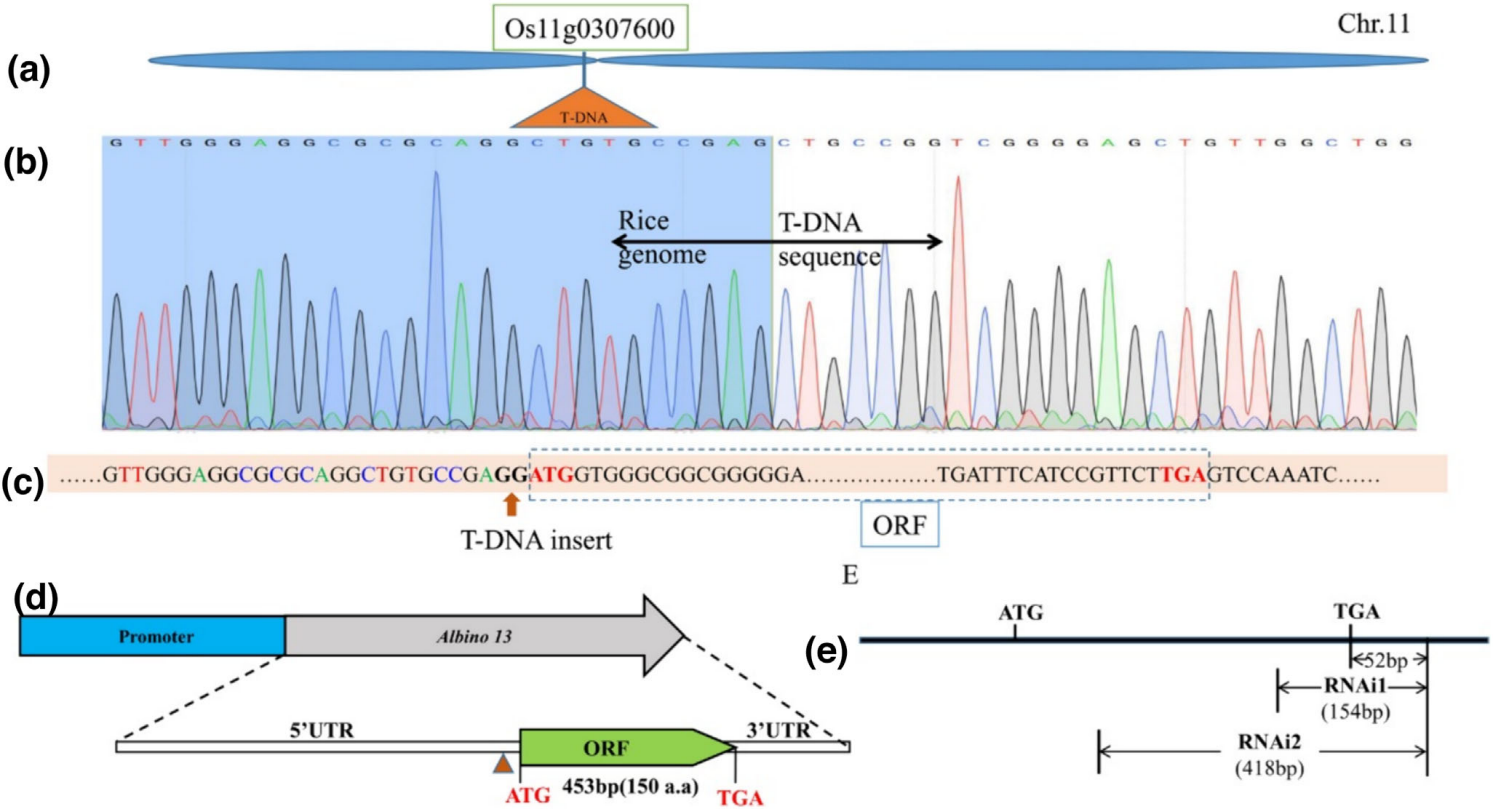
Positional cloning of the *OsAL13* gene. T‐DNA was inserted into Os11g0307600 at the short arm of chromosome 11 in rice. (a) The OsAL13 locus was narrowed to a 21‐kb region on Os11g0307600. (b) The sequence peak chart of T‐DNA insertion; the vertical division line indicates the point of T‐DNA insertion. (c) T‐DNA inserts before the base G of promoter ATG. (d) Structure of *OsAL13*, with ATG and TGA representing the start and stop codons, respectively. Blue boxes indicate the promoter, and green boxes indicate the open reading frame (ORF). (e) Two different fragments for RNAi construction are shown as sketch maps.

Genetic analysis indicated that the *osal13* mutant phenotype could be subject to control by a single recessive gene, as evidenced by the 3:1 segregation ratio between WT phenotypes and *Osal13* MT in the T_2_ population (162 albino plants, 513 normal plants, χ^2^ = .1961). By the DNA‐walking technique, a specific amplified fragment, including the fragment of CaMV3′UTR, was obtained. The BLAST search with the flanking sequence indicated that the T‐DNA was inserted into the *OsJNBa0005p21* clone of rice chromosome 11 (Figure 2a,b; Figure S1), which contained 21 predictive genes. The T‐DNA was inserted into the third gene, *Os11g0307600*. A 662‐bp‐specific fragment was obtained by amplification of the specific primers OsAL13‐1Fa and OsAL13‐1Ra using LiyuB cDNA as a template. Sequencing results of the target band showed that there was a 453‐bp ORF, which was identified as the target gene *OsAL13* (Figure 2d). The T‐DNA was inserted before the base G of the start codon ATG (Figure 2c), causing the start codon to be misread and resulting in an albino phenotype. To investigate potential regulatory cis‐acting elements, we analyzed the promoter region of *OsAL13* using PlantCARE—this detailed analysis revealed that it contained 19 different regulatory elements involved in light responsiveness, hormone responsiveness, transcription, and drought responsiveness (Table S1). Among them, nine regulatory cis‐acting elements are involved in light responsiveness. The *OsAL13* may have related light‐response functions.

### 3.3 Suppression of *OsAL13* leads to an osal13‐like phenotype

**FIGURE 3:**
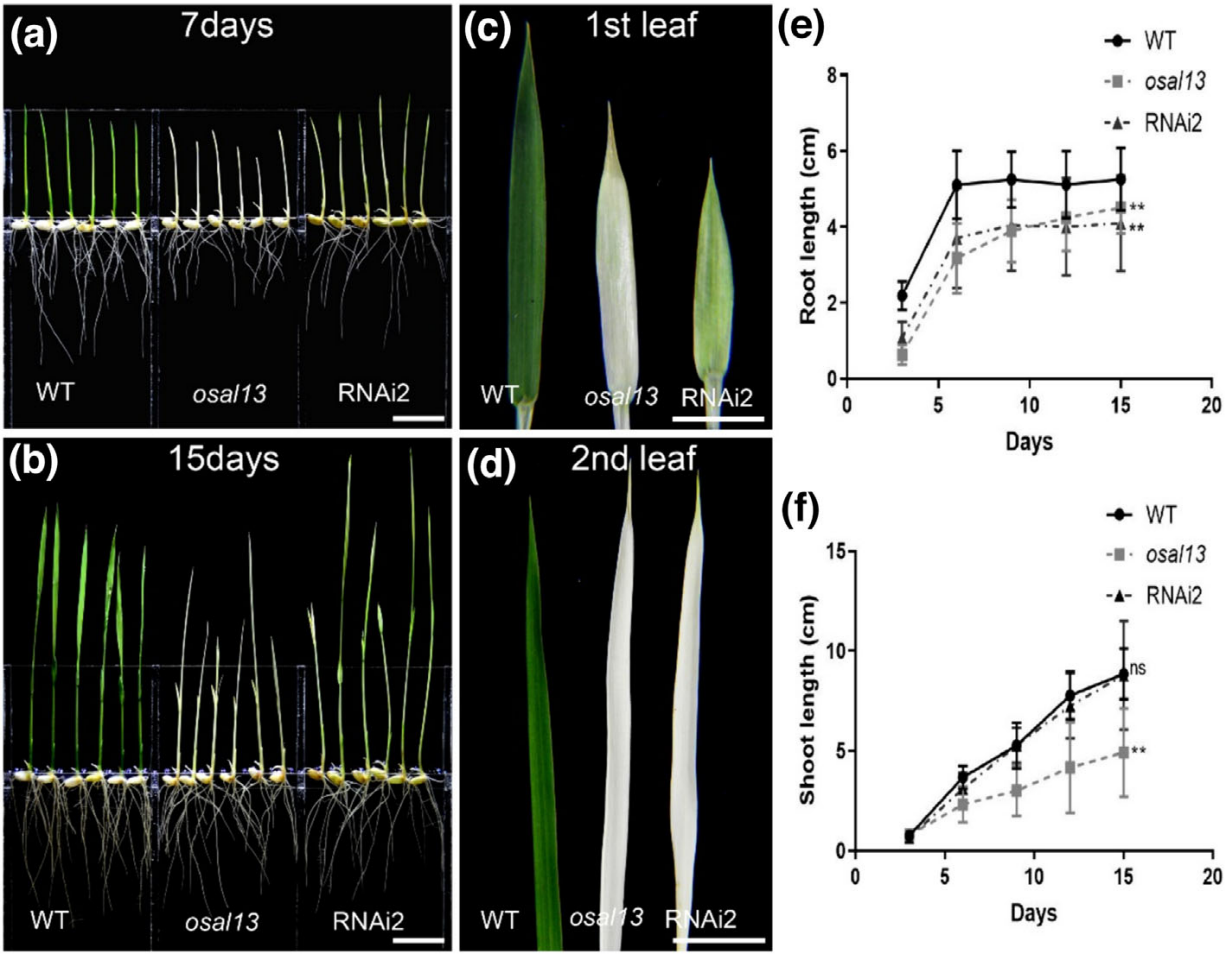
The different phenotypes of the wild type (WT), *osal13* mutant, and RNAi2. At 7 days after germination (DAG) (a) and 15 DAG (b). Bars = 1 cm. The comparison of the first (c) and second leaf (d) of the WT, *osal13* mutant, and RNAi2. Bars = 1 cm. Seedling growth determination as root length (e) and shoot length (f), with data points representing the mean ± SD.

Suppression of *OsAL13* resulted in lethality of seeding, which caused difficulty in further study of the *OsAL13* functions. To address this, we produced weak *osal13* alleles by specific knockdown of *OsAL13* expression. Two 154‐ and 418‐bp WT genomic DNA fragments of *Os11g0307600*, containing part of the coding sequences and part of the 3′UTR (52 bp) (Figure 2e), were cloned in a binary vector to generate the constructs of RNAi1 and RNAi2 and then transformed into WT (LiyuB). Finally, 22 independent RNA interference lines (RNAi1 and RNAi2) were obtained with different expression levels of *OsAL13*. Of these 22 independent RNAi lines, RNAi2–3, RNAi2–6, and RNAi2–8, with the most significant down‐regulation of *OsAL13*, showed a highly similar phenotype to that of the *osal13* mutant (Figure 3a,b). The first and second leaves of WT, *osal13* mutant, and RNAi2 plants were observed at the seedling stage. The first leaf of the WT was normal green, the *osal13* mutant was completely albino, and RNAi2 was yellow‐green (Figure 3c). The second leaf of the WT was green, but the *osal13* mutant and RNAi2 were white overall (Figure 3d). The shoot and root lengths of WT, *osal13* mutant, and RNAi2 plants were measured. Root lengths of both *osal13* mutant and RNAi2 plants were significantly inhibited compared with WT (Figure 3e). The albino *osal13* mutant significantly inhibited shoot length. Down‐regulation of *OsAL13* significantly inhibited root length and seedling growth (Figure 3f). These results further confirmed that *OsAL13* was the candidate gene and was required for chloroplast development, and disruption of its function resulted in the albino leaf phenotype.

### 3.4 Subcellular localization and expression pattern of *OsAL13* in different tissues

**FIGURE 4:**
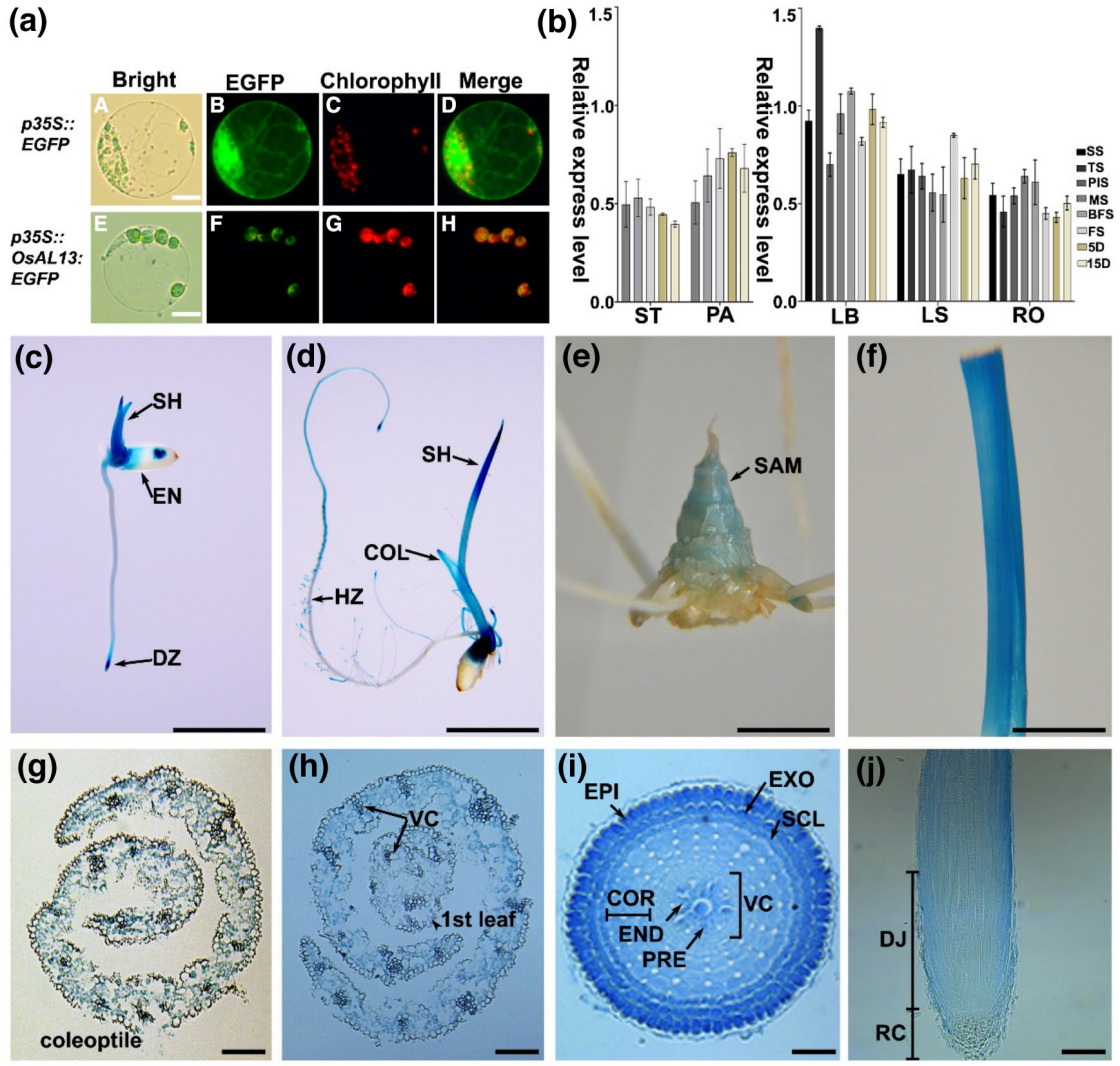
Subcellular location and expression pattern of *OsAL13 in different tissues*. (a) Analysis of the subcellular localization of the OsAL13 protein in rice protoplasts. Green fluorescence shows green fluorescent protein. Chloroplast autofluorescence is shown in red. Scale bars are 50 μm. (b) Quantitative real‐time polymerase chain reaction analysis of *OsAL13* in comparison with the rice β‐actin gene. *OsAL13* expression was detected in the panicle (PA), stem (ST), leaf blade (LB), leaf sheath (LS), and root (RT) of the rice plant in the seedling stage (SS), tiller stage (TS), panicle initiation stage (PIS), meiotic division phase of the rice panicle (flower) development (MS), before the flowering stage (BFS), flowering stage (FS), 5 days after pollination (5 D), and 15 days after pollination (15 D) of the rice plant. (c, d) β‐Glucuronidase (GUS) staining of seeds at 2 days after germination (DAG) and 5 DAG. Histochemical staining showing *OsAL13* expression in the cell division zone (DZ), endosperm (EN), shoot (SH), root hair zone (HZ), and coleoptile (COL). (e) GUS staining of the stem tip at the tillering stage of the shoot apical meristem (SAM). (f) GUS staining of the leaf at the meiotic phase of the flag leaf. GUS staining of the cross‐sections from 5 DAG in the leaf primordia (g) and (h) as well as root tip (i) and (j). COR, cortex; END, endodermis; EPI, epidermis; EXO, exodermis; PER, pericycle; SCL, sclerenchyma; VC, vascular cylinder.

To investigate the subcellular localization of OsAL13 protein, the protoplasts of transgenic plants *p35S::EGFP* and *p35S::OsAL13:EGFP* were extracted. The green fluorescence signal was apparent over the whole cell in the EGFP‐positive control (Figure 4a, A–D; Figure S2). In *p35S::OsAL13:EGFP*, the green fluorescence signal co‐localized with chloroplast autofluorescence (Figure 4a, E–H), suggesting that the target gene was expressed in the chloroplast. This result supported the view that *OsAL13* plays an important role in regulating chloroplast development.

Different tissues from different growth stages of LiyuB were assayed for temporal and spatial expression patterns of *OsAL13* using qRT‐PCR (Figure 4b). Transcripts of *OsAL13* were detected in all examined tissues and were most abundant in the leaf blade and weakest in the stem (Figure 4b).

We next examined the expression of the *GUS* gene under the control of the *OsAL13* promoter from LiyuB in transgenic plants. All tissues exhibited GUS activity, and the GUS signal was particularly strong in the root tip (Figure 4c,d) and shoot apical meristem (Figure 4e). During leaf development, reporter genes were mainly expressed in young leaves (Figure 4f). The *GUS* gene was expressed in the coleoptile and the first leaf of the bud sheath and strongly expressed in the vascular sheath (Figure 4g,h). The GUS staining of the root tip at 4 DAG was performed by frozen section, and the target gene was mainly expressed in root epidermis, exodermis, and sclerenchyma cells (Figure 4i) and in the meristem and growth region (Figure 4j). Longitudinal sectioning of the bud sheath showed that the target gene was mainly expressed in the meristem region and undeveloped leaves; the target gene was expressed in all cells, especially leaf sheath cells. Our results suggested that *OsAL13* is constitutively expressed in a variety of tissues and primarily plays a role in green tissues and in places where cell growth is active.

### 3.5 *OsAL13* regulates the related development of the photosynthetic system

**FIGURE 5:**
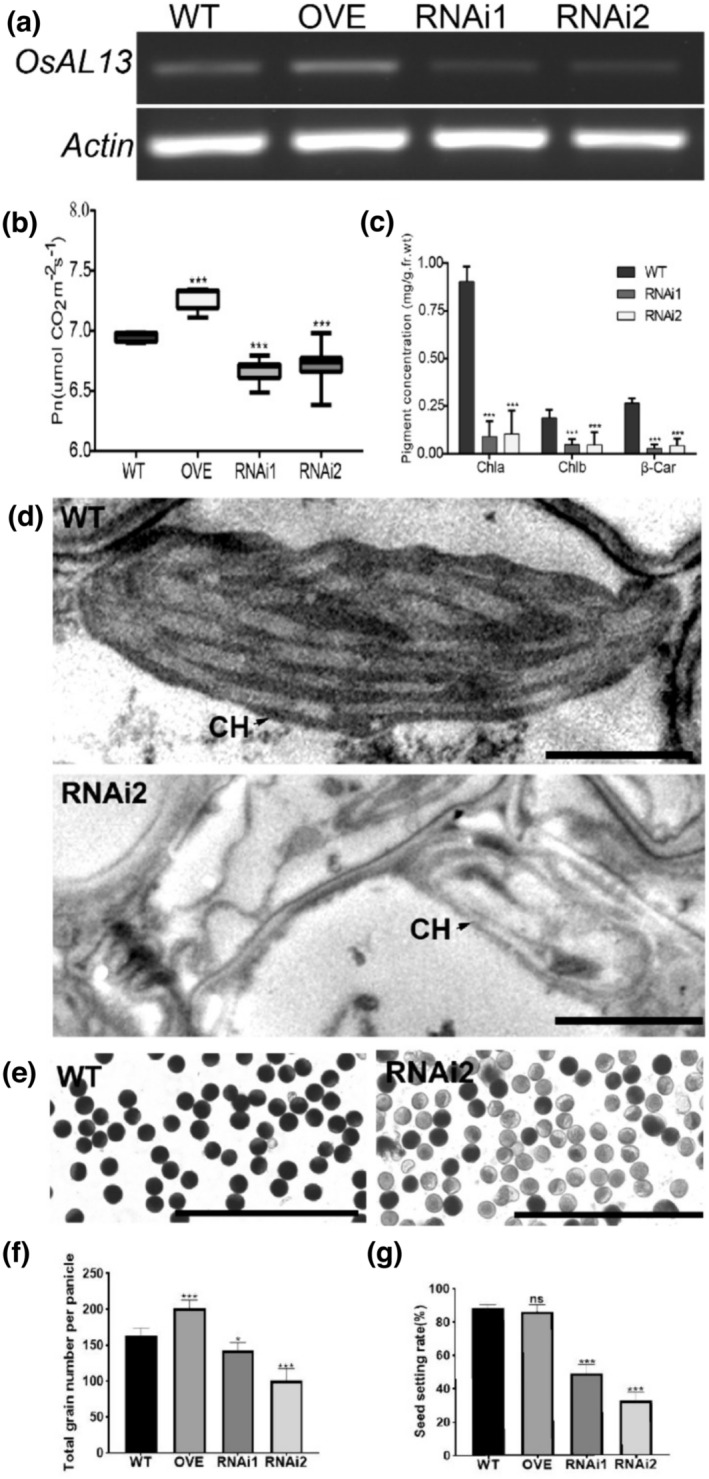
Comparison of the morphology and internal structure of wild‐type (WT), overexpressing (OVE), and RNAi lines. (a) Expression analysis of *OsAL13* in seedings at 7 days after germination (DAG) of the WT, *OsAL13* OVE, RNAi1, and RNAi2 lines by quantitative real‐time polymerase chain reaction. (b) Photosynthetic rate of WT, OVE, RNAi1, and RNAi2. (c) Determination of pigment contents in leaves of WT, RNAi1, and RNAi2. Leaf samples were collected from 20‐day‐old WT, RNAi1, and RNAi2 plants grown in a tank containing nutrient solution. Car, carotenoid; Chla, chlorophyll a; Chlb, chlorophyll b. Transmission electron microscopy observations of the third‐leaf sections of WT and RNAi2. The ultrastructure of chloroplasts of the WT is typical. The chloroplast structure is complete, the matrix is dense, and the grana lamellae are arranged along the long axis of the chloroplast. (d) The thylakoid structure of WT is clear, whereas the shape of the chloroplast of RNAi2 became irregular, and the whole chloroplast shows a vesicle structure. Scale bars are 10 μm. (e) Pollen viability assay by I2‐KI in WT and RNAi2 lines. Scale bars are 200 μm. Analysis of total grain number per panicle (f) and seed setting rate (g) of WT, OVE, and RNAi lines. Significant differences are indicated by asterisks (***p < .001).

The qRT‐PCR analysis of WT, OVE, RNAi1, and RNAi2 plants indicated that transcript levels of *OsAL13* were increased in OVE and partially eliminated in RNAi1 and RNAi2 (Figure 5a).

We further determined photosynthetic rates in WT, OVE, and RNAi plants. The RNAi lines showed significantly lower photosynthetic rates, but the OVE plants showed significantly higher photosynthetic rates compared with WT plants (Figure 5b). Because the mutant exhibited an albino phenotype, we speculate that the target gene might affect chlorophyll development. Therefore, disrupting the expression of the target gene might lead to abnormal chloroplasts. The chlorophyll content was very much higher in WT than in RNAi1 and RNAi2 plants, and carotenoids were almost undetectable in RNAi lines (Figure 5c). We infer that inhibition of *OsAL13* expression affects the development of chlorophyll and carotenoids.

To further explore the regulation function of *OsAL13* in chloroplast development, we compared the chloroplast ultrastructure of RNAi and WT plants. As expected, WT cells contained normal chloroplasts and had good lamellar structure, normal stacked grana, and thylakoids. In contrast, most RNAi cells had predominantly undeveloped chloroplasts, and these were small. They also lacked thylakoids with a grouped or layered structure, and some chloroplasts showed internal vacuolation (Figure 5d).

### 3.6 Suppression of *OsAL13* expression results in decreased pollen fertility and seed setting rates

Pollen fertility of WT, OVE, and RNAi2 was 94.0 ± 2.3%, 98.8 ±0.63%, and 14.0 ± 3.4%, respectively, the pollen fertility of RNAi1 was 0. Compared with the WT, the pollen fertility of RNAi1 and RNAi2 plants was significantly reduced (Figure 5e). And then, the total grain number per panicle of OVE plants was significantly increased, but that of RNAi plants was significantly reduced (Figure 5f). The seed setting rate of RNAi plants was also significantly reduced (Figure 5g). Therefore, the total grain number per panicle and seed setting rate of RNAi2 were significantly lower. The other yield‐related agronomic traits of WT and transgenic RNAi plants were measured (Table 2). After *OsAL13* expression was inhibited, plant height was reduced with less effect on effective panicle number, panicle length, number of primary branches, number of secondary branches, and grain length, but the effects on grain length and grain thickness were greater. Combined with the previous pollen fertility, we speculated that the decrease in pollen fertility could lead to a decrease in seed setting rate.

**TABLE 2:** Analysis of yield‐related characters (per plant) among wild‐type and *OsAL13*‐RNAi plants.

Traits	Wild‐type	*AL13*‐RNAi1	*AL13*‐RNAi2
Plant height (cm)	87.40 ± 3.10	80.20 ± 1.70*	68.50 ± 3.50***
Tillering numbers	4.40 ± 1.10	4.60 ± 1.50	5.80 ± 1.80
Panicle length (cm)	20.10 ± 1.60	16.10 ± 1.60***	18.80 ± 2.00
No. of primary branches	10.00 ± 2.30	8.90 ± 2.30	9.90 ± 1.50
No. of secondary branches	21.20 ± 6.50	13.10 ± 7.00	20.40 ± 5.70
Grain length (mm)	7.03 ± .25	7.09 ± .09	7.00 ± .07
Grain width (mm)	3.16 ± .15	2.97 ± .13***	2.96 ± .11***
Grain thickness (mm)	2.25 ± .07	2.11 ± .07***	2.07 ± .07***

* *p* < .05.

*** *p* < .001.

### 3.7 *OsAL13* affects hormone levels in plant tissues and alters the expression of chloroplast‐associated genes

**FIGURE 6:**
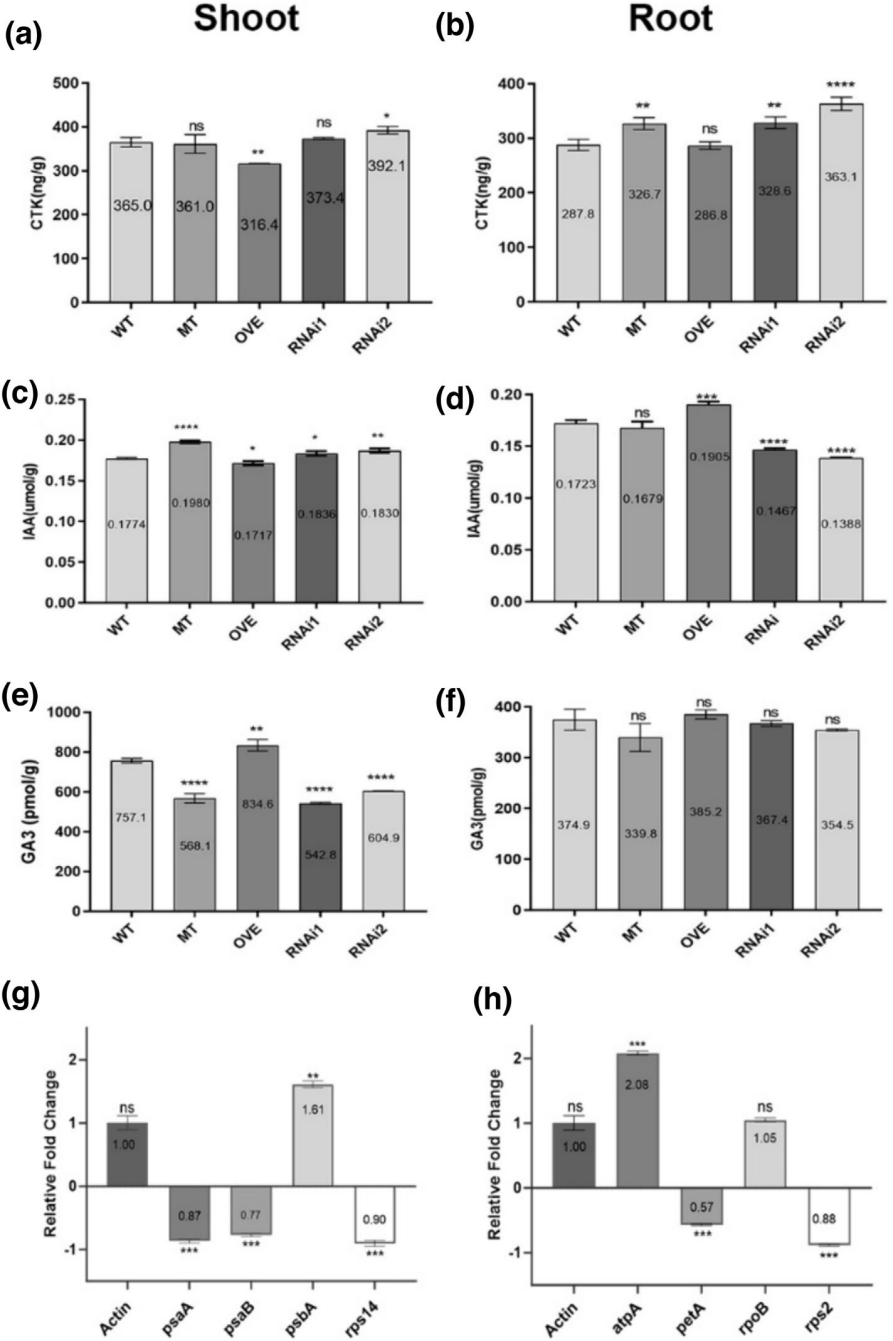
*OsAL13* control of hormone distribution and expression of chloroplast‐associated genes. Hormone content comparison among wild‐type (WT), *osal13* mutant, overexpressing (OVE), and *OsAL13*‐RNAi plants at the seedling stage. Contents of cytokinin (CTK), indole‐3‐acetic acid (IAA), and gibberellin A3 (GA3) in shoot (a, c, e) and root (b, d, f). Values are mean ± SD. Asterisks indicate significant differences: *p < .05, **p < .01, and ***p < .001. The relative fold change of the chloroplast‐associated genes, including plastid‐encoded polymerases (g) and nucleus‐encoded polymerases (h), by quantitative real‐time polymerase chain reaction.

The endogenous hormones IAA, GA3, and CTK were measured in the shoots and roots of WT, *osal13* mutant, OVE, and RNAi plants (Figure 6). In the shoots, the CTK contents of RNAi2 were significantly increased. The IAA contents of the MT and RNAi lines were significantly higher than those of WT, but the IAA contents of OVE were significantly lower than those of WT. However, the GA3 contents have an opposite trend with IAA in the shoots of MT, RNAi, and OVE (Figure 6a,c,e). The contents of CTK in the shoots of OVE were significantly decreased, while in the shoots of RNAi2, they were significantly increased compared with WT. CTK contents in the roots of MT and RNAi were higher than those in WT, while there were no significant differences in CTK contents between OVE and WT. The contents of IAA in the root of OVE have higher levels; while the MT roots were not significantly different, the RNAi roots have lower levels compared with WT. The contents of GA3 in the roots of MT, OVE, and RNAi were not significantly different compared with WT (Figure 6b,d,f). Thus, *OsAL13* overexpression led to enhanced IAA levels in the roots and increased GA3 levels in the shoot.

In higher plants, chloroplast genes are transcribed by two types of RNA polymerases: nucleus‐encoded polymerases (NEPs) and plastid‐encoded polymerases (PEPs). To confirm the role of *OsAL13* in the biological process of chloroplast development, multiple chloroplast‐associated genes were analyzed by qRT‐PCR. In the *osal13* mutant, the expression levels of three PEP genes (*psaA*, *psaB*, and *rps14*) were down‐regulated, whereas those of the other (*psbA*) P and NEP were deficient in the *osal13* mutant and led to defective chloroplast biogenesis.

## 4 DISCUSSION

Using a DNA‐walking cloning method, we isolated the gene *OsAL13*, which encodes a novel protein. We found that *OsAL13* is a novel gene in rice that has not been cloned or studied. The protein sequence encoded by *OsAL13* is shown in Figure S3. Searching for the conserved domain of the protein encoded by *OsAL13* in the NCBI was not successful (Figure S4). Homologous genes and homologous proteins were found, but homologous proteins (genes) with known functions were also not found (Figure S5).

In the *OsAL13* mutations, the most obvious phenotypic feature was albino lethality. In previous studies, the gene that caused the albino lethality phenotype basically affected chloroplast development and chlorophyll metabolism abnormalities (Hayashi‐Tsugane et al., 2014; Jiang et al., 2014; Zeng et al., 2017). The rice albinic seedling mutants *Oslas1*, *Osasl4* (Zhou et al., 2021), *Osra1* (Zheng et al., 2019), and *OsCpn60β1* (Wu et al., 2020) also showed albino phenotypes and eventually died, and chlorophyll contents were significantly reduced. Similarly, chlorophyll a and chlorophyll b of the *osal13* mutant were significantly lower than those of WT. The RNAi tests verified that *OsAL13* was responsible for the albino phenotype (Figure 3a,b). Hence, *OsAL13* is essential for chloroplast development.

Our results suggest that *OsAL13* was constitutively expressed and had a high level of transcription in leaves, suggesting that *OsAL13* may play an important role in leaf development. Subcellular localization in rice protoplasts showed that the OsAL13 protein was located in chloroplasts (Figure 4a). Further research showed that the pigment concentration of RNAi plants decreased significantly (Figure 5b,c), and the chloroplast structure was not complete, especially because there was a lack of neatly stowed thylakoid structure (Figure 5e). Meanwhile, the photosynthetic rate of the OVE plant was highest, so the total grain number per panicle of the OVE plant would be significantly increased. The thylakoid is important for the synthesis of photosynthetic pigment and the fixation of light energy by photosynthesis. Functional chloroplasts are the main sites of photosynthesis, providing both a relatively independent space environment and key proteins for their successful operation, thus establishing the photosynthetic autotrophic conditions of plants (Emanuelsson et al., 1999). Therefore, *OsAL13* is a necessary gene for chloroplast development.

It is well known that a chloroplast develops from a simple and undeveloped proplastid during light‐dependent differentiation (Adam et al., 2011). The chloroplast is a semi‐autonomous organelle containing about 100 genes, although more than 3000 proteins function within it (Leister, 2003). Thus, nuclear‐encoded factors play essential roles in regulating chloroplast development, which requires the coordinated expression of both nucleus and chloroplast‐encoded genes. The *osal13* mutant disrupts the transcripts of plastid and nuclear genes associated with chloroplast development (Figure 6). The transcript accumulation of both PEP‐ and NEP‐dependent genes (*PetA* and *rps2*) and PEP‐transcribed plastid genes (*psaA* and *psaB*) was severely suppressed (Figure 6g,h), suggesting that the accumulation of transcripts for PEP components did not result in the formation of functional PEP due to the disruption of the transcription/translation apparatus.

Carotenoids are a class of isoprene compounds synthesized in collaboration with chlorophyll and play many important roles in all photosynthetic organisms (Rodríguez‐Villalón et al., 2009). For example, in addition to stabilizing membranes (including thylakoid membranes) and promoting photomorphogenesis, many carotenoid molecules also act as auxiliary pigments, assisting chlorophyll to capture light energy and transfer it to the photosystem, from which excess energy can be transferred, reducing oxidative stress in the photosystem (Meier et al., 2011). Therefore, a lack of carotenoid protection during chloroplast development may cause metabolic disorders such as the accumulation of reactive oxygen species and abnormal energy metabolism. In this study, carotenoids were nearly undetectable in the *osal13* mutant, and whether this will lead to plant metabolic disorders, the accumulation of reactive oxygen species, and abnormal energy metabolism requires further exploration.

In this study, expression of endogenous hormones showed that, compared with WT, GA3 content significantly rose in shoots of OVE plants but significantly declined for *osal13* mutant and RNAi shoots. Previous studies indicated that GA3 in Arabidopsis promotes chloroplast cell division and cell expansion of chloroplasts (Hussain et al., 2003). The CTK has a positive effect on chloroplast development. In Arabidopsis, bud removal activates CTK signaling mediated by Arabidopsis response modulator B, induces chlorophyll accumulation and photosynthetic remodeling, and promotes chloroplast formation in isolated root cells (Kobayashi et al., 2017). In rice, GA3 controls cell division by stimulating the expression of some cyclins and cyclin‐dependent kinase (CDK) genes to activate G1/S and G2/M transitions (Sauter, 1997). Therefore, we speculate that *OsAL13* may regulate chloroplast proliferation through endogenous plant hormones.

This study illustrated the defects in chloroplast growth and development observed in the *osal13* mutant, characterized by a noticeable albino phenotype in leaves that ultimately resulted in seedling lethality. Molecular cloning revealed *OsAL13* as a novel nuclear recessive gene, consistently expressed in various tissues and localized within chloroplasts. Additionally, RNAi‐mediated knockdown and overexpression of *OsAL13* affirmed its indispensable role in chloroplast development.

## AUTHOR CONTRIBUTIONS

Lijuan Chen and Dongsun Lee conceived the study and supervised the experiments; Xiaoqiong Guo, Chunli Wang, and Qian Zhu performed the experiments and produced the figures; Wenhua Dongchen, Xiaoling Zhang, Wei Li, and Hui Zhang participated in performing the experiments; Mengting Li, Zar Ni Naing Nant Nyein, and Cui Zhang analyzed the data; and Xiaoqiong Guo and Chunli Wang wrote the manuscript. All authors have read and agreed to the published version of the manuscript.

## CONFLICT OF INTEREST STATEMENT

The authors report no conflicts of interest.

## PEER REVIEW

The peer review history for this article is available in the Supporting Information for this article.

## Supporting information


**Data S1.** Peer Review.

**Figure S1.** BLAST search with the flanking sequence of T‐DNA.
**Figure S2.** Analysis of the subcellular localization of the OsAL13 protein in rice protoplasts.
**Figure S3.** Protein sequence encoded by *OsAL13*. The red ATG is the start codon, the red TGA is the stop codon, the underlined is the CDS region of the target gene and the corresponding amino acid sequence.
**Figure S4.** OsAL13 protein conserved domain query in NCBI. No conserved domains were identified for this query sequence.
**Figure S5.** *OsAL13* homologous gene search. No homologous genes of known function were found.
**Table S1.** Cis‐acting element prediction of *OsAL13* promoter region.

## Data Availability

All the data for this article are available in the manuscript.
