# Supplementary material for: Albino lethal 13, a chloroplast‐imported protein required for chloroplast development in rice
Source: Plant Direct. 2024 Jun 20;8(6):e610. doi: 10.1002/pld3.610 (PMC11189691; doi:10.1002/pld3.610)
Supplement: Supplementary file 2 — Figure S1. BLAST search with the flanking sequence of T‐DNA. Figure S2. Analysis of the subcellular localization of the OsAL13 protein in rice protoplasts. Figure S3. Protein sequence encoded by OsAL13. The red ATG is the start codon, the red TGA is the stop codon, the underlined is the CDS region of the target gene and the corresponding amino acid sequence. Figure S4. OsAL13 protein conserved domain query in NCBI. No conserved domains were identified for this query sequence. Figure S5. OsAL13 homologous gene search. No homologous genes of known function were found. Table S1. Cis‐acting element prediction of OsAL13 promoter region. [file PLD3-8-e610-s002.docx]

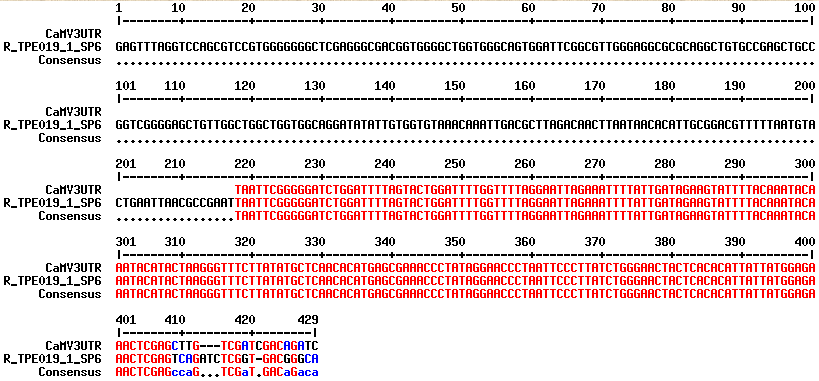
**Supplementary Material**

**Figure S1.** BLAST search with the flanking sequence of T-DNA.


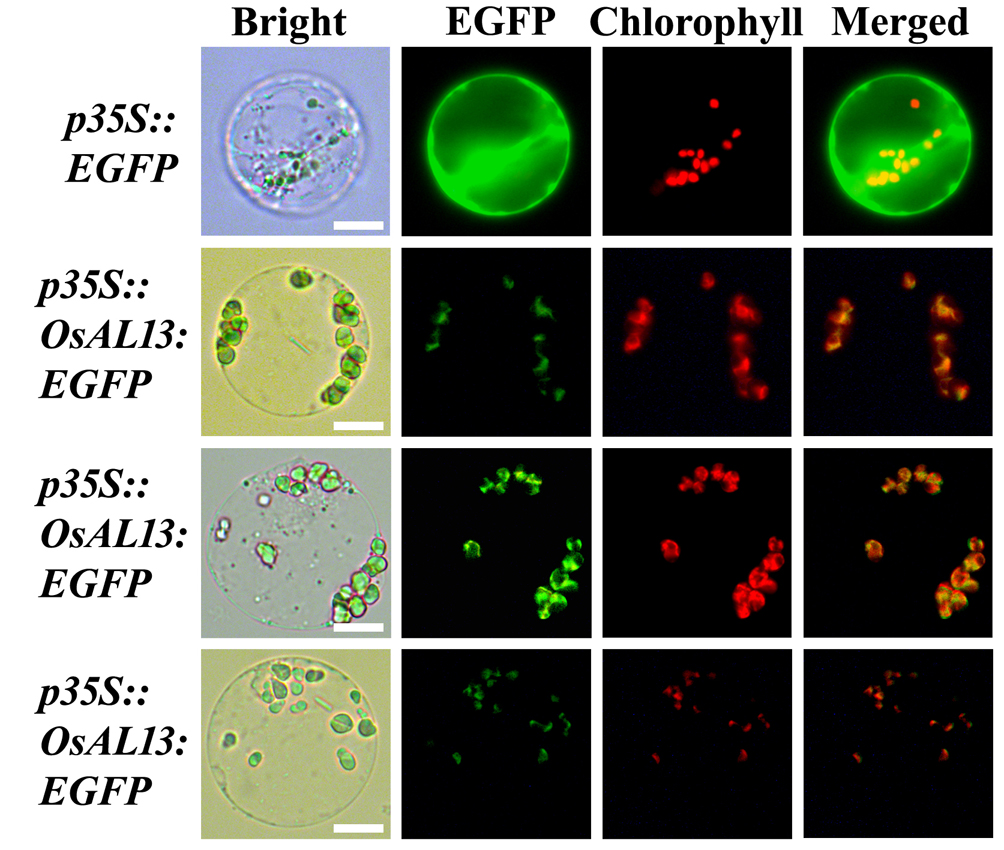


**Figure S2.** Analysis of the subcellular localization of the OsAL13 protein in rice protoplasts.


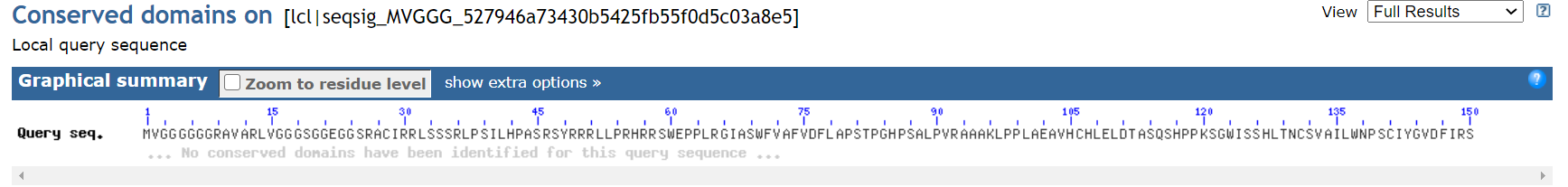
**
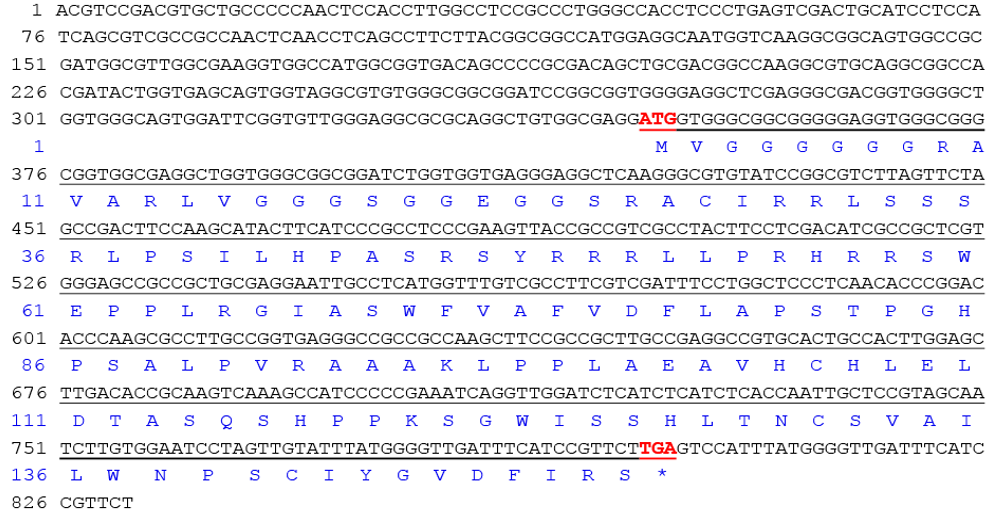
Figure S3.** Protein sequence encoded by *OsAL13*. The red ATG is the start codon, the red TGA is the stop codon, the underlined is the CDS region of the target gene and the corresponding amino acid sequence.


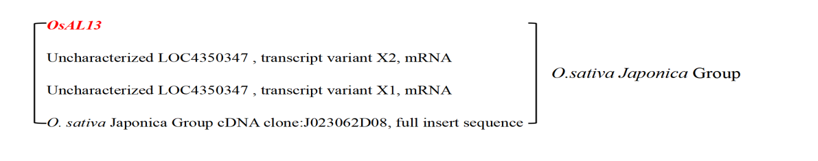
**Figure S4.** OsAL13 protein conserved domain query in NCBI. No conserved domains were identified for this query sequence.

**Figure S5.** *OsAL13* homologous gene search. No homologous genes of known function were found.

**Table S1.** Cis-acting element prediction of *OsAL13* promoter region.

| **Site name** | **Core sequence** | **Type** | **Function** |
| --- | --- | --- | --- |
| GATA-motif | GATAGGA | light responsive element | part of a light responsive element |
|  | AAGGATAAGG |  | part of a light responsive element |
| I-box | gGATAAGGTG |  | part of a light responsive element |
| G-Box | CACGTG |  | cis-acting regulatory element involved in light responsiveness |
| GT1-motif | GGTTAAT |  | light responsive element |
|  | GGTTAA |  | light responsive element |
| Sp1 | GGGCGG |  | light responsive element |
| LAMP-element | CCTTATCCA |  | part of a light responsive element |
| G-box | CACGTC |  | cis-acting regulatory element involved in light responsiveness |
|  | CACGTG |  | cis-acting regulatory element involved in light responsiveness |
|  | CACGAC |  | cis-acting regulatory element involved in light responsiveness |
|  | CACGTC |  | cis-acting regulatory element involved in light responsiveness |
| TCCC-motif | TCTCCCT |  | part of a light responsive element |
|  | TCTCCCT |  | part of a light responsive element |
| Box 4 | ATTAAT |  | part of a conserved DNA module involved in light responsiveness |
| ABRE | ACGTG | hormone responsive | cis-acting element involved in the abscisic acid responsiveness |
|  | CACGTG |  | cis-acting element involved in the abscisic acid responsiveness |
|  | ACGTG |  | cis-acting element involved in the abscisic acid responsiveness |
|  | ACGTG |  | cis-acting element involved in the abscisic acid responsiveness |
| CGTCA-motif | CGTCA |  | cis-acting regulatory element involved in the MeJA-responsiveness |
| TCA-element | CCATCTTTTT |  | cis-acting element involved in salicylic acid responsiveness |
| TATC-box | TATCCCA |  | cis-acting element involved in gibberellin-responsiveness |

| **Table S1.** Cis-acting element prediction of *OsAL13* promoter region. Continued Table S1 | | | |
| --- | --- | --- | --- |
| **Site name** | **Core sequence** | **Type** | **Function** |
| TATA-box | TATAAGAA | transcription | core promoter element around -30 of transcription start |
|  | TATATA |  | core promoter element around -30 of transcription start |
|  | ATATAT |  | core promoter element around -30 of transcription start |
|  | ccTATAAAaa |  | core promoter element around -30 of transcription start |
|  | TATAAA |  | core promoter element around -30 of transcription start |
|  | TATAA |  | core promoter element around -30 of transcription start |
| CAAT-box | CAAAT |  | common cis-acting element in promoter and enhancer regions |
| MBS | CAACTG | drought responsive | MYB binding site involved in drought-inducibility |
| CCAAT-box | CAACGG |  | MYBHv1 binding site |
| TC-rich repeats | GTTTTCTTAC |  | cis-acting element involved in defense and stress responsiveness |
| Box III | atCATTTTCACt |  | protein binding site |
