# Supplementary material for: Albino lethal 13, a chloroplast‐imported protein required for chloroplast development in rice
Source: Plant Direct. 2024 Jun 20;8(6):e610. doi: 10.1002/pld3.610 (PMC11189691; doi:10.1002/pld3.610)
Supplement: Supplementary file 1 — Data S1. Peer Review. [file PLD3-8-e610-s001.pdf]

Plant Direct
Open Access

Albino lethal 13, a chloroplast imported protein required for chloroplast development in rice

Xiaoqiong Guo [1,6*], Chunli Wang [1*], Qian Zhu [1,2,3], Wenhua Dongchen [4], Xiaoling Zhang [5], Wei Li [6], Hui Zhang[4], Cui Zhang [1], Zar Ni Naing Nant Nyein [1], Mengting Li [1], Lijuan Chen [1, 2, 3#], Dongsun Lee[1, 2, 3#]

Decision Letter Round 1:

May 13, 2024

Prof. Lijuan Chen
Rice Research Institute, Yunnan Agricultural University
Rice Research Institute, College of Agronomy and Biotechnology
Kunming, Yunnan 650201
China

RE: Albino lethal 13, a chloroplast imported protein required for chloroplast development in rice

Dear Dr. Chen:

Thank you for submitting to Plant Direct. All required reviews have been returned and we have now finished our evaluation of your manuscript. In light of the reviewers' and editor's comments, further revisions are needed before the paper can be accepted for publication in Plant Direct.

Please view the editors' and reviewers' comments below and use their suggestions as a guide while you work on your revision.

When uploading the revised version of this article, please be sure to include the following:

-A word document that contains your response to the reviewers. You should respond to each reviewer comment and note the changes made to the manuscript. If you do not agree with a reviewer's comment and choose not to make a suggested revision, please explain why. Please try to provide as complete an answer as possible to each reviewer's criticisms.
-A tracked changes document with each change highlighted
- A clean version of the latest version of the manuscript

To upload your revision, please click the link below.
https://plantdirect.msubmit.net/cgi-bin/main.plex?el=A7Lr6gn5A6lka1I2A9ftdaEW9CFBAEHk8wWBZg8VdgZ

In order to provide as timely a service as possible, we ask that your revision is resubmitted within three

months after receipt of this request. If an extension is needed, please send a request, along with a brief explanation, to the editorial office at plantdirect@wiley.com .

Please note that, in addition to publishing reviewer comments, the author's responses to review comments will also be published alongside the final version of the paper. If you would not like the author's responses to be published, please contact the editorial office at plantdirect@wiley.com .

Thank you very much for giving us an opportunity to review your work. I look forward to receiving the next version.

Sincerely,

Ana Fortes

Ana Fortes

Editor, Plant Direct

-- Editor comments:

-- Reviewer comments:
Reviewer #1:

This is an excellent manuscript. Please see attached for some typo correction.

In the conclusion you may mention some specific function of the gene and some other functions (putative) for future work.

May elaborate on the homologous sequences found for the gene

May touch on endosymbiosis in the discussion section

The gene is located in the genomic DNA and it is obvious but you may mention it.

Reviewer #2:

The manuscript "Albino lethal 13, a chloroplast imported protein required for chloroplast development in rice" by Guo et al. cloned and characterized the chloroplast located gene, Albino and lethal 13. This study demonstrated that albino and lethal 13 play a key role in rice development and RNAi lines display decreasing pollen fertility and albino leaf phenotype. T-DNA mutants display albino phenotype and

seedling lethality whereas overexpression lines have higher seeds setting and increased level of hormones.

Minor errors

1. Page 1, In the Abstract information, Line 7, the authors mentions that no homologous gene or known conserved domain was found. However, in the discussion section, page 10, line 348-349 "Homologous genes and homologous proteins were found", this can lead to confusion and contradiction between two sections, please correct either one of the statements.

2. Page 12, Line 427-429, Acknowledgment section, sentence fragment, should put a comma before, the Applied basic research in Yunnan Province and the NSF of China

3. Page 12, Line 439, REFERANCES, spelling error, should be REFERENCES

Other questions and concerns

1. Did the authors have any speculations in the molecular mechanism that resulted in poor fertility issue in RNAi lines?

2. How do the authors rule out the possibility of multiple insertion of T-DNA in different locations that lead to potential albino phenotype?

3. Did the overexpression of the albino and lethal 13 plants have any detectable leaf development or color phenotype that result in higher photosynthetic rate?

4. For the accumulation of reactive oxygen species in al13 mutant, did the authors perform related experiment, including examine the expression level of hydrogen peroxide scavenger related enzyme, including APX and other enzymatic components like superoxide dismutase (SOD) and catalase (CAT)?

Decision Letter Round 2:

May 18, 2024
Prof. Lijuan Chen
Rice Research Institute, Yunnan Agricultural University
Rice Research Institute, College of Agronomy and Biotechnology
Kunming, Yunnan 650201
China

MSID: 2024-01455R1
MS TITLE: Albino lethal 13, a chloroplast imported protein required for chloroplast development in rice

Dear Dr. Lijuan Chen:

I am pleased to inform you that your manuscript "Albino lethal 13, a chloroplast imported protein

required for chloroplast development in rice" has been accepted for publication in Plant Direct.

Your article will appear online in the next available issue of Plant Direct. To ensure your article gets published as quickly as possible, please pay attention to the steps detailed below. We have found that most of the delays happen at this stage, especially at the payment stage, so please respond as quickly as possible when prompted.

License Agreement: Once your article has been accepted it will move to Production and undergo admin and file checking - you may receive an email with any queries we have at this stage. When all required items are received by the publisher and queries resolved, the corresponding author will receive an email from Wiley's Author Services system which will ask them to log on at https://authorservices.wiley.com/bauthor and will present them with the appropriate license for completion. Your article cannot be published until both the signed license agreement and payment of the article fee have been received.

Payment of the Open Access Article Publication Fee: All articles published in Plant Direct are fully open access: immediately and freely available to read, download and share. Plant Direct charges a publication fee to cover the publication costs. The corresponding author for this manuscript should have already received a quote from the payments team (cs-openaccess@wiley.com) with the estimated article publication fee; please email cs-openaccess@wiley.com if this has not been received. The corresponding author should log on to the Wiley Author Services site, where the publication fee can be paid by credit card or an invoice. Pro Forma can also be requested. Payment of the publication charge must be received before the article will be published online.

Proofs: You will have the opportunity to look over your paper once more when you receive the author proofs for your article. The proofs will be with you in approximately two weeks. Please note that, in addition to publishing reviewer comments, the author's responses to review comments will also be published alongside the final version of the paper. If you would not like the author's responses to be published, please contact the editorial office at plantdirect@wiley.com .

Promotion of your article: You can help your research get the attention it deserves! Check out Wiley's free Promotion Guide for best-practice recommendations for promoting your work at www.wileyauthors.com/eeo/guide. And learn more about Wiley Editing Services which offers professional video, design, and writing services to create shareable video abstracts, infographics, conference posters, lay summaries, and research news stories for your research at www.wileyauthors.com/eeo/promotion.

Thank you again for your contribution to Plant Direct. If you have any questions, feel free to contact the editorial office at plantdirect@wiley.com .

Sincerely,

Ana Fortes

Ana Fortes

Plant Direct Open Access

Editor, Plant Direct

Author Response:

Editor, Plant Direct

Subject: Plant Direct: 2024-01455: Decision Letter

Dear Ana Fortes,

We are extremely thankful to you for giving us a chance to submit the revised version of the manuscript entitled "Albino lethal 13, a chloroplast imported protein required for chloroplast development in rice". We are also thankful to the reviewers for their valuable comments. The manuscript has been revised and modified according to the reviewers' comments. Please find attached the modified version of the manuscript and the point by point response to the reviewers' comments.

I hope you will find the revised version satisfactory.

Reviewer 1 comments：

Comment 1. This is an excellent manuscript. Please see attached for some typo correction.

Response: Thank you so much. As suggested by the reviewer, the sentence has been corrected.

Comment 2. In the conclusion you may mention some specific function of the gene and some other functions (putative) for future work.

Response: We found this gene possible function on cytogenesis involving to tetraploids for example, but the data is still not enough for this part. And we will continue this part in next paper.

Comment 3. May elaborate on the homologous sequences found for the gene

Response: We designed primers based on possible predicted sequence and that primer did not obtain results for homologous sequences. In this study, the sequence (supplementary material Figure 3) is coming from mRNA and used for cloning work. Based on this sequence, we designed the OVE and RNAi vectors.

Comment 4. May touch on endosymbiosis in the discussion section

Response: Previously, we did not consider this aspect, and we plan to explore it further in our future work, which will be detailed in our upcoming paper. Thank you for your suggestion.

Comment 5. The gene is located in the genomic DNA and it is obvious but you may mention it.

Response: The gene is situated on chromosome 11 (Figure 2). We conducted sequencing and inserted the T-DNA at the designated position, followed by cloning of the cDNA. *OsAL13* has been successfully cloned from the wild-type (WT) and employed in our investigation.

Reviewer 2 comments：

The manuscript "Albino lethal 13, a chloroplast imported protein required for chloroplast development in rice" by Guo et al. cloned and characterized the chloroplast located gene, Albino and lethal 13. This study demonstrated that albino and lethal 13 play a key role in rice development and RNAi lines display decreasing pollen fertility and albino leaf phenotype. T-DNA mutants display albino phenotype and seedling lethality whereas overexpression lines have higher seeds setting and increased level of hormones.

Minor errors

Comment 1. Page 1, In the Abstract information, Line 7, the authors mentions that no homologous gene or known conserved domain was found. However, in the discussion section, page 10, line 348-349 "Homologous genes and homologous proteins were found", this can lead to confusion and contradiction between two sections, please correct either one of the statements.

Response: As suggested by the reviewer, we have been corrected the discussion section at page 10, line 348-349.

Comment 2. Page 12, Line 427-429, Acknowledgment section, sentence fragment, should put a comma before, the Applied basic research in Yunnan Province and the NSF of China.

Response: As suggested by the reviewer, we have been modified the Acknowledgment section.

Comment 3. Page 12, Line 439, REFERANCES, spelling error, should be REFERENCES

Response: Page 12, Line 439, Spelling of “REFERANCES” has been corrected “REFERENCES”

Other questions and concerns

Comment 4. Did the authors have any speculations in the molecular mechanism that resulted in poor fertility issue in RNAi lines?

Response: We hypothesize that this gene is required for chloroplast development in rice. Consequently, we anticipate a potential reduction in photosynthetic ability in RNAi plants. Additionally, we expect a subsequent decrease in fertility.

Comment 5. How do the authors rule out the possibility of multiple insertion of T-DNA in different locations that lead to potential albino phenotype?

Response: In our study, the population of T_1 generation follow the "Mendelian Law" and show 3:1 segregation ratio and indicated that a single recessive gene control. DNA-walking was performed three times to find the target region and the same result were come out.

Comment 6. Did the overexpression of the albino and lethal 13 plants have any detectable leaf development or color phenotype that result in higher photosynthetic rate?

Response: Actually, the overexpression transgenic plants show tetraploids and diploid. In this paper, we focus on diploid plants from the overexpression transgenic line, which display a slightly darker leaf colour compared to the wild type. Tetraploid research remains a subject for our future studies.

Comment 7. For the accumulation of reactive oxygen species in al13 mutant, did the authors perform related experiment, including examine the expression level of hydrogen peroxide scavenger related enzyme, including APX and other enzymatic components like superoxide dismutase (SOD) and catalase (CAT)?

Response: Before we have not considered to focus on the accumulation of reactive oxygen species in al13 mutant, so we are extremely thankful to your kindly advise. Next study will continue to do this part including examine the expression level of hydrogen peroxide scavenger related enzyme, including APX and other enzymatic components like superoxide dismutase (SOD) and catalase (CAT).

Besides these modifications, any other minor changes/modifications suggested by the reviewer have been done and can be tracked in the track changes version of the manuscript.

Yours sincerely,

Li Juan Chen (Corresponding Author); Dong Sun Lee (Corresponding Author)
Professor; Professor
Rice Research Institute, Yunnan Agricultural University, 650201, Kunming Yunnan, China.
E-mail: 964136487@qq.com; dongeast@naver.com
Telephone:（+86）13099983337;18508865980
